# Low-Dose Albendazole Inhibits Epithelial-Mesenchymal Transition of Melanoma Cells by Enhancing Phosphorylated GSK-3*β*/Tyr216 Accumulation

**DOI:** 10.1155/2021/4475192

**Published:** 2021-12-20

**Authors:** Zhiqiang He, Shun Lei, Fucheng Liang, Liuchang Tan, Weinan Zhang, Luoyingzi Xie, Hong Zheng, Yuangang Lu

**Affiliations:** ^1^Department of Plastic & Cosmetic Surgery, Army Medical Center of PLA, Amy Medical University, Chongqing 400042, China; ^2^Department of Oncology and Southwest Cancer Center, Southwest Hospital, Army Medical University, Chongqing 400038, China; ^3^The Institute of Immunology, Army Medical University, Chongqing 400038, China; ^4^Department of Thoracic Surgery, Xinqiao Hospital, Amy Medical University, Chongqing 400037, China

## Abstract

Albendazole (ABZ) is an effective broad-spectrum anthelmintic agent that has been widely used for humans and animals. Previous studies have reported that ABZ exhibits antitumor effects against melanoma and other different cancer types; however, it is unknown whether ABZ exerts the inhibitory effect against melanoma metastasis. In this study, we aimed to investigate the inhibitory effect of ABZ on melanoma cells. Through in vitro studies, we discovered that low-dose ABZ treatment significantly inhibited the migration and invasion, but not the proliferation, of A375 and B16-F10 cells in a dose-dependent manner. Further analysis revealed that ABZ treatment reduced the expression level of snail family transcriptional repressor 1 (Snail) in the cytoplasm and nucleus by decreasing the levels of phosphorylated AKT (pAKT) Ser473/GSK-3*β* (pGSK-3*β*) Ser9 and increasing pGSK-3*β*/Tyr216, resulting in a significant upregulation of E-cadherin and downregulation of N-cadherin and ultimately reversing the epithelial-mesenchymal transition (EMT) process of melanoma cells. In contrast, the continuous activation of AKT via transfected plasmids elevated the protein levels of pAKT Ser473/pGSK-3*β* Ser9 and Snail and antagonized the inhibitory action of ABZ. We also confirmed that ABZ treatment effectively inhibited the lung metastasis of melanoma in nude mice in vivo. Subsequent immunohistochemical analysis verified the decreased pAKT Ser473/pGSK-3*β* Ser9 and increased pGSK-3*β*/Tyr216 levels in ABZ-treated subcutaneous tumors. Therefore, our findings demonstrate that ABZ treatment can suppress the EMT progress of melanoma by increasing the pGSK-3*β*/Tyr216-mediated degradation of Snail, which may be used as a potential treatment strategy for metastatic melanoma.

## 1. Introduction

Melanoma is a highly malignant tumor derived from melanocytes that mostly occurs in the skin but can also be found in the mucous membrane and internal organs. As one of the deadliest skin cancers [1], melanoma incidence continues to increase worldwide [2] at a rate of 3% every year [3]. Furthermore, approximately 230,000 people are diagnosed with primary melanoma worldwide, which causes almost 55,500 deaths annually [4]. Local melanoma can be effectively treated by surgical resection in the early stage, with a five-year survival rate of >90% [5]. In contrast, metastatic melanoma has a poor prognosis, with a five-year survival rate of <10%. Hence, surgical resection is only appropriate for early local melanoma and is almost ineffective after metastases occur [6].

Previous studies have reported that the high mortality rate of malignant melanoma is significantly related to its high metastasis rate [7]. Since melanoma is an epithelial malignant tumor, the epithelial-mesenchymal transition (EMT) process plays a vital role in melanoma metastasis [8]. EMT is a necessary procedure for normal embryonic development and damage repair [9]; however, it also occurs during malignant tumor progression [10]. The EMT process enables the migration and invasion of epithelial cell-derived malignant tumor cells, which is accompanied by mesenchymal-like changes [11], including loss of cell polarity, decreased intercellular adhesion, and altered expression of cell surface proteins [12]. The abnormal expression of specific markers is a typical feature of the EMT process, such as the upregulation of N-cadherin, snail family transcriptional repressor 1 (Snail), Vimentin, Occludin, and fibronectin 1 (FN1) and the downregulation of E-cadherin [13], which ultimately leads to melanoma metastasis [14]. As an important transcription factor in the EMT process, Snail, which can move into the cell nucleus after phosphorylated at Ser246 and determine the relative expression of downstream N- and E-cadherin proteins [15, 16], is directly regulated by the upstream signaling pathway RAC-alpha serine/threonine-protein kinase (AKT)/Glycogen synthase kinase-3 beta (GSK-3*β*) during tumor progression [17, 18]. GSK-3*β*/Tyr216 (active form) can mediate nucleus export and degradation of Snail, while GSK-3*β*/Ser9 (inactive form) phosphorylated by pAKT can maintain its stability. Therefore, the change in the ratio of Tyr216/Ser9 could be very important for the level of Snail and the ability of tumor cells to invade and metastasize [16, 18, 19]. Although recent advances have been made in cancer medicine, immunotherapy, and targeted drug discovery, the rapid occurrence of drug resistance in cancer patients usually leads to disease recurrence [20]. At present, there is no long-term and effective treatment method for melanoma metastasis. Therefore, identifying the potential inhibitors for EMT is required for the suppression of tumor progression and the development of therapeutic interventions for melanoma [8].

Recently, drug repositioning has become an increasingly attractive option for medical treatments due to the pharmacokinetics and safety of existing drugs, which can reduce the difficulty and risk of clinical trials to a certain extent [21, 22]; in addition, this strategy has economic advantages compared to complete new drug development [23]. One example is albendazole (ABZ), a kind of benzimidazole derivative and antiparasitic drug with known antitumor properties that can be repositioned as an anticancer drug [22, 24]. Despite preexisting researches regarding that ABZ may have an antimelanoma effect by inhibiting proliferation [25] or promoting apoptosis [26], it is still unknown whether ABZ can also exhibit inhibitory effects against the metastasis of melanoma. In this study, we aimed to determine the effects of ABZ treatment on the migration and invasion of A375 and B16-F10 melanoma cells during EMT and to identify associated signaling pathways and potential targets for future studies.

## 2. Materials and Methods

### 2.1. Cell Lines and Culture

The A375 human melanoma and B16-F10 mouse melanoma cell lines were obtained from American Type Culture Collection (ATCC; Manassas, VA, USA), while B16-F10-luciferase (B16-F10-luc) cells were purchased from Shanghai Model Organisms Center (Shanghai, China). The A375 and B16-F10-luc cells were cultured in Dulbecco's modified Eagle's medium-high glucose (SH30243.01, HyClone, Logan, UT, USA) containing 10% fetal bovine serum (FBS; SH30084.03, HyClone), 1% penicillin/1% streptomycin (P1400, Solarbio, Beijing, China), 1% L-glutamine (G0200, Solarbio), and 1% nonessential amino acid (N1250, Solarbio). The cells were cultured in an incubator at 37°C with 5% CO_2_ (Forma 3111, Thermo Fisher Scientific, Waltham, MA, USA).

### 2.2. Plasmid Construction

The human AKT1 coding sequence was amplified and cloned into pcDNA3.1 (+) plasmids. The constitutively active AKT plasmid (CA-AKT; T308D/S473D) was obtained by mutating the wild-type human AKT1 using QuikChange^®^ Site-Directed Mutagenesis Kit (Stratagene, La Jolla, CA, USA) and then validated by sequencing. Endotoxin-free CA-AKT plasmids (used for transfection) were extracted using Endo-Free Plasmid Midi Kit-fast (Omega Bio-tek, Norcross, GA, USA). GSK-3*β*/Tyr216 wild-type (WT) and GSK-3*β* Y216F mutant pLenti plasmid which encodes WT or Y216F cDNA were purchased from Obio Technology Co. Ltd (Shanghai, China). VSV-G plasmid pMD2.G and psPAX2 packaging plasmids were gifts from Prof. Wenyue Xu (Army Medical University).

### 2.3. Cell Transfection and Transduction

The A375 and B16-F10 melanoma cells with approximately 80% confluency were selected for transfection. Opti-MEM™ medium (Gibco, Thermo Fisher Scientific Waltham, MA, USA) was used to dilute the Lipofectamine™ 3000 reagent (L3000008, Thermo Fisher Scientific, Waltham, MA, USA) and plasmid master mix containing plasmids and P3000™ reagent, which were subsequently mixed according to the manufacturer's instructions and added to the cell culture medium. HEK293T cells (donated by the Laboratory of Thoracic Surgery, Xinqiao Hospital, Army Medical University) were transfected with the GSK-3*β* wild-type (WT) or GSK-3*β* Y216F pLenti plasmid combined with pMD2.G and psPAX2 plasmids for collecting the lentiviruses by using Lipofectamine 3000 (L3000008, Thermo Fisher Scientific, Waltham, MA, USA) according to the manufacturer's instructions. GSK-3*β* wild-type A375 melanoma cells and GSK-3*β*/Y216F A375 melanoma cells were generated by using lentiviral transduction and by selection in presence of blasticidin (5ug/ml) (ant-bl-05, InvivoGen, San Diego, USA).

### 2.4. Cell Viability and Proliferation Assay

ABZ (HY-B0223, MCE, Monmouth Junction, NJ, USA) was dissolved in dimethyl sulfoxide (DMSO; final concentration <0.1%) to a final concentration of 10 mmol/L and stored for subsequent use. The cell counting kit-8 (CCK-8; C0037, Beyotime, Shanghai, China) assay was used to analyze cell viability and proliferation. Melanoma cells in the logarithmic growth phase (5 × 10^3^ cells) were seeded and cultured in 96-well plates containing 100 *μ*L medium. The experimental groups were treated with DMSO and ABZ at different concentrations (0.1, 0.2, 0.4, 0.8, 1.6, 3.2, 6.4, and 12.8 *μ*M), while the blank groups were untreated. After 24 h, 10 *μ*L CCK-8 reagent was added to each well, and the plates were incubated at 37°C for 2 h. The absorbance values were subsequently measured at 490 nm using a microplate reader (Beckman Coulter, Brea, CA, USA).

### 2.5. Wound Healing Assay

A wound-healing test was used to detect the effect of ABZ treatment on the migration of melanoma cells. The A375 and B16-F10 cells were inoculated in 6-well plates with a culture medium containing 10% FBS. After the cell coverage reached 95%, a T-1000-B pipette tip was used to scratch a wound on the cells. Then, the plate was washed twice with phosphate-buffered saline (PBS; ZLI-9061, ZSGB-Bio, Beijing, China) to remove the floating cells, and cell images were randomly taken using an inverted microscope (Leica inverted microscope DMi8, Wetzlar, Germany). Finally, the cells were treated with ABZ at different concentrations (0.1, 0.2, and 0.4 *μ*M) for 24 h, and cell images were obtained again using the same microscope. ImageJ software (NIH, Bethesda, MD, USA) was used to quantify the wound healing area on the cell images.

### 2.6. Transwell Invasion Assay

The bottom of the transwells with 8 *μ*m pores (662638, Greiner Bio-One, Frickenhausen, Germany) was precoated with Matrigel (356234, Corning, Bedford, MA, USA). The A375 and B16-F10 cells were starved using a medium without FBS for 12 h. The cells (6 × 10^4^ per well) were inoculated into a serum-free medium containing different concentrations of ABZ (0.1, 0.2, and 0.4 *μ*M) and allowed to invade through the 8 *μ*m pores into the lower chambers containing medium with 10% FBS. After 24 h, the transwells were fixed with 4% paraformaldehyde (P0099, Beyotime) for 20 min and stained with 0.1% crystal violet (HY-B0324A, MCE) for another 20 min. Images of five randomly selected positions were obtained by using Leica inverted microscope DMi8 and analyzed using ImageJ software (NIH) to determine the number of cells that invaded the lower chambers.

### 2.7. Western Blotting

A375 and B16F10 cells were treated with 0.4 *μ*M ABZ alone for 24 h or per-transfected with CA-AKT or pcDNA3.1 (+) plasmids and treated with ABZ for 24 h; for proteinase inhibiting, A375 cells were cotreated with ABZ and 10 *μ*M MG132 for 24 h; GSK-3*β*/WT and GSK-3*β*/Y216F stable expressing A375 cells were treated with 0.4 *μ*M ABZ alone for 24 h. Then, these cells were lysed in RIPA buffer (89900, Thermo Fisher Scientific, Waltham, MA, USA) to extract the total protein. Nuclear and cytoplasmic proteins were extracted separately using NE-PER^™^ Nuclear and Cytoplasmic Extraction Reagents (78833, Thermo Fisher Scientific, Waltham, MA, USA) and quantified using BCA Protein Detection Kit (P0012s, Beyotime). The protein samples were resolved by 10% sodium dodecyl sulfate-polyacrylamide gel electrophoresis (1610183, Bio-Rad Laboratories, Hercules, CA, USA) and then transferred to polyvinylidene fluoride (PVDF; IPFL00010, Merck KGaA, Darmstadt, Germany) membranes using electroporation fluid. The membranes were blocked with No protein blocking solution (C510042-0500, Sangon Biotech, Shanghai, China) for 1 h at 25°C. After washing thrice with Tris-buffered saline with Tween^®^20 (TBST; B548105-0500, Sangon Biotech), the blocked PVDF membranes were cut into appropriate shapes, incubated with the primary antibodies, and kept in a shaker overnight at 4°C. The primary antibodies, namely, anti-phospho-AKT (Ser473) rabbit antibody (44-623G), anti-AKT (44-609G), anti-N-cadherin (13-2100), anti-E-cadherin (14-3249-82), anti-Snail (14-9859-82), anti-Vimentin (MA5-16409), anti-Occludin (33-1500), anti-FN1 (PA5-29578), *β*-actin (MA5-15452), anti-GSK3B (phospho-Tyr216) rabbit antibody (44-604G), anti-GSK3B (phospho-Ser9) rabbit antibody (MA5-38235), anti-GSK-3*β* rabbit antibody (PA5-95845), anti-SNAI1 (phospho-Ser246) rabbit antibody (PA5-37739), and proliferating cell nuclear antigen (PCNA; PA5-27214), were purchased from Thermo Fisher Scientific. Subsequently, the PVDF membranes were incubated with the corresponding secondary antibodies (ZB-2306, ZSGB-Bio) for 1 h. After washing thrice with TBST, the protein samples were visualized using Fusion Solo S Chemiluminescence Imaging System (VILBER, Collégien, France). The obtained western blot data were analyzed using ImageJ software (NIH).

### 2.8. Real-Time Reverse Transcription Quantitative PCR (RT-qPCR)

Total RNA was extracted from melanoma cells using TRIzol^®^ reagent (15596026, Thermo Fisher Scientific, Waltham, MA, USA) and reverse-transcribed into cDNA using PrimeScript RT reagent Kit with gDNA Eraser (RR047A, Takara, Kyoto, Japan), according to the manufacturer's instructions. Real-time RT-qPCR analysis was performed to investigate the differences in the mRNA expression of EMT-related genes before and after ABZ treatment. The PCR reaction cocktail (25 *μ*L) contained 0.5 *μ*L each of forward and reverse primers, 12.5 *μ*L SYBR^®^ Pre-mix Ex Taq TM II (RR820A, Takara), 10.5 *μ*L double-distilled water, and 1 *μ*L cDNA. The PCR cycling conditions were as follows: 95°C for 15 s, 60°C for 30 s, and amplification for 40 cycles. Each experiment was performed thrice. The relative expression levels were calculated using the 2^−ΔΔCt^ method, with *β-*actin as the internal control for normalization. The primers used for E-cadherin, N-cadherin, snail, Vimentin, Occludin, FN1, and *β-*actin are listed in Supplementary Table 1.

### 2.9. Animal Experiments

The nude mice (4 weeks old) used for animal experiments were obtained from the Animal Experiment Center of the Army Medical Center of PLA (Chongqing, China) and maintained in the SPF animal house. All studies were conducted in accordance with the experimental protocol approved by the Ethical Review of Experimental Animals of the Army Medical Center of PLA. First, B16-F10-luc cells (5 × 10^5^) were injected through the caudal vein. Second, after 1 week, the mice were anesthetized and injected with Xenolight^™^ D-Luciferin Potassium Salt (Perkin Elmer, Boston, MA, USA), 5 min later, they were put on the imaging plate, the exposure time was 20 s, and the unit of measurement is photon flux, and in vivo fluorescence images were obtained using IVIS Lumina III Imaging System (Perkin Elmer). Third, after confirming that the tumor model was successfully constructed, the mice were randomly divided into three groups, namely, 50 mg/kg, 20 mg/kg ABZ, and control, the experimental groups used PBS with different concentrations of ABZ (containing 0.5% sodium carboxymethyl cellulose + 2% DMSO), and the control groups used PBS (containing 0.5% sodium carboxymethyl cellulose + 2% DMSO), the dosage was 100 *μ*L, and they were treated once daily via intragastric administration. Fourth, after continuous intragastric gavage for 10 days, in vivo fluorescence images were obtained again using the same method. Finally, the mice were dissected, and the lungs were taken out and washed with PBS. The melanoma nodules on the lung surfaces were counted and fixed with 4% paraformaldehyde.

### 2.10. Hematoxylin-Eosin (HE) Staining

Following conventional methods, the lung tissue was embedded in paraffin and cut into 4 *μ*m sections for HE staining. The sections were stained with hematoxylin staining solution for 3 min, washed with distilled water for 15 s, and stained with 1% hydrochloric acid and ethanol for 15 s. After washing with distilled water for 1 min, the sections were stained with eosin for 50 s and washed again with distilled water for 15 s. The sections were dehydrated by gradient ethanol, soaked in xylene, and finally sealed with neutral balsam.

### 2.11. Immunohistochemistry (IHC) Assay and Scoring

IHC detection of key EMT proteins was performed using the Dako Envision FLEX + system (Dako, Berlin, Germany). Paraffin sections were deparaffinized. Antigen retrieval was performed by heating the samples in citrate buffer (pH 6.0) in the microwave for 15 min, then returning the samples to room temperature, followed by washing with phosphate-buffered saline (PBS). The samples were blocked with the Dako REAL Peroxidase-Blocking Solution for 15 min. The slides were incubated at 4°C overnight with the rabbit antibodies for anti-N-cadherin, Rabbit ant-E-cadherin, anti-phospho-AKT (Ser473), anti-GSK3B (phospho-Tyr216), and anti-GSK3B (phospho-Ser9), followed by incubation (30 min) with the secondary antibody. Slides were stained with 3,3′-diaminobenzidine tetrahydrochloride (DAB) for 2 min. The percentages of cells that were positive for the markers were scored as follows: 0–5%, no positive cells; 1, <25% positive cells; 2, 25–50% positive cells; 3, 50–75% positive cells; 4, 75–100% positive cells. The staining intensity was scored as follows: 0, no positive cells; 1, weak staining; 2, moderate staining; and 3, strong staining. The immunohistochemical staining score was obtained by multiplying the percentage score by the intensity (0, 1, 2, 3, 4, 6, 8, 9, or 12). The X-tile v3.6.1 statistical package [27] was used to analyze the IHC assay results.

### 2.12. Statistical Analysis

The data were expressed as means ± standard deviation (SD) of three independent experiments. One-way ANOVA was used for comparison of two groups if the data were normally distributed, and if not, the Mann–Whitney *U* test was used. The Tukey-Kramer statistic was used for multiple comparisons in normally distributed; if not, Dunnett's test was used. Statistical Analysis was performed by using SPSS 25.0 statistical software (IBM SPSS).

## 3. Results

### 3.1. Low-Dose ABZ Treatment Significantly Inhibits the Migration and Invasion of Melanoma Cells In Vitro

**Figure 1:**
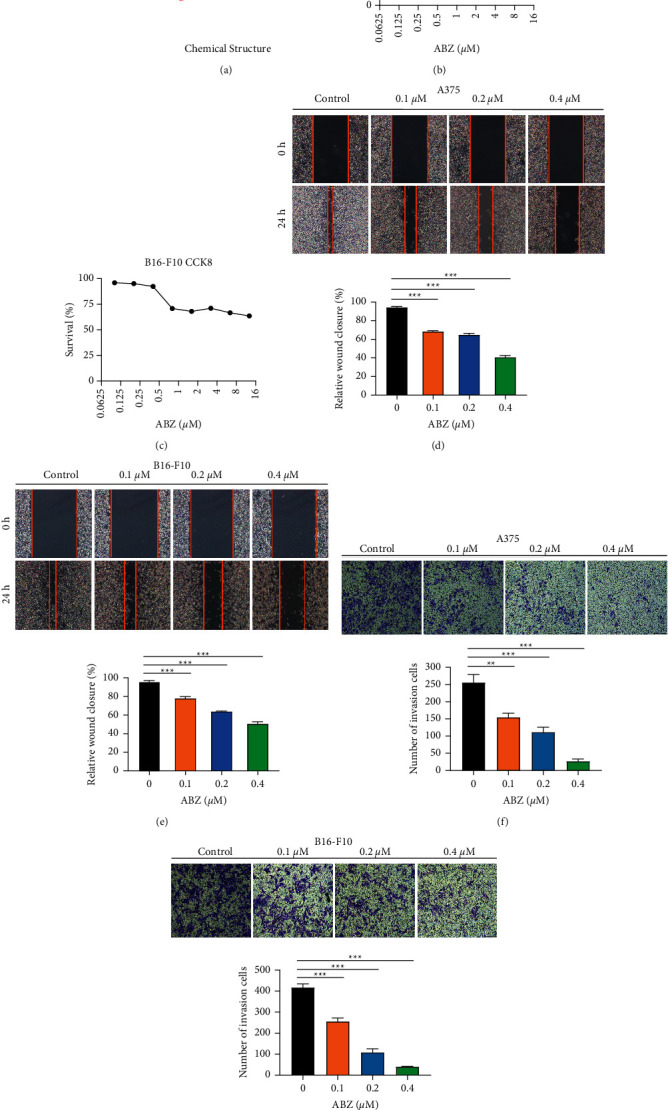
Low-dose albendazole (ABZ) treatment effectively inhibits the migration and invasion of melanoma cells. (a) The chemical structure of ABZ. (b-c) The A375 and B16-F10 cells were treated with or without different concentrations of ABZ. CCK-8 analysis was performed to detect cell viability and proliferation. (d–e) The A375 and B16-F10 cells were treated with different concentrations of ABZ (0.1, 0.2, and 0.4 *μ*M). The wound healing area and relative wound closure rate (%) in melanoma cells were measured and analyzed 24 h after treatment. (f-g) The inhibitory effect of ABZ on the invasion of A375 and B16-F10 cells was detected using transwell experiments. The data are expressed as means ± SD. All experiments were performed thrice. ^*∗∗*^*P* < 0.01; ^*∗∗∗*^*P* < 0.001.

ABZ is a benzimidazole carbamate drug (Figure 1(a)). To determine the effect of ABZ on the migration and invasion abilities of melanoma cells, we performed CCK-8 assays on melanoma cells treated with ABZ at different concentrations and discovered that low-dose ABZ (<0.4 *μ*M/L) treatment had no significant effect on the proliferation of A375 and B16-F10 cells (Figures 1(b) and 1(c)). However, the wound healing assays showed that low-dose ABZ treatment for 24 h significantly inhibited the migration of both A375 (Figure 1(d)) and B16-F10 (Figure 1(e)) cells in a dose-dependent manner. Furthermore, the Matrigel invasion assay revealed that low-dose ABZ treatment markedly suppressed the invasion abilities of the two melanoma cell lines in a dose-dependent manner (Figures 1(f)–1(g)). These findings suggest that low-dose ABZ can significantly inhibit the migration and invasion, but not the proliferation, of melanoma cells in vitro.

### 3.2. Low-Dose ABZ Treatment Suppresses the Lung Metastasis of Melanoma Cells In Vivo

**Figure 2:**
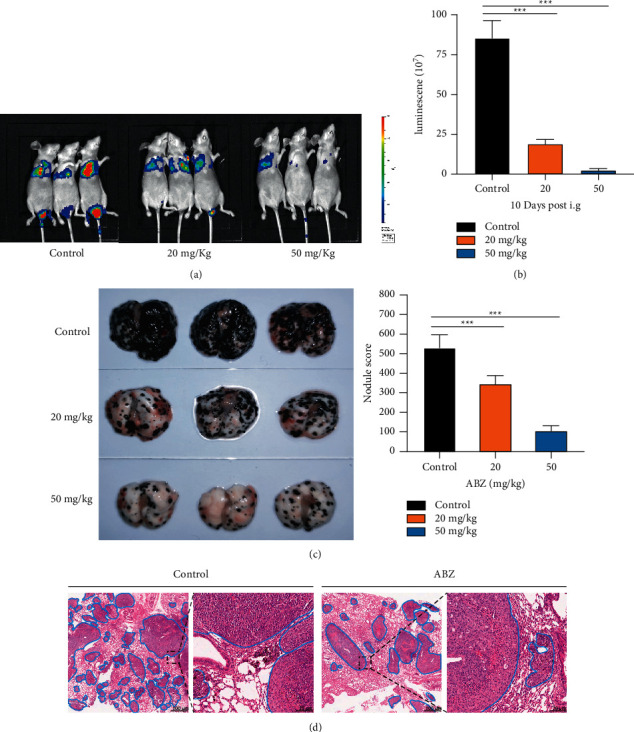
ABZ treatment suppresses lung melanoma metastasis in mice. (a) Representative image and (b) statistical analysis of luminescence intensity in B16-F10-luc tumor-bearing nude mice treated with different concentrations of ABZ (*n* = 5). (c) The upper, middle, and lower panels represent the melanoma nodules on the lung surfaces of mice in the control, 20 mg/kg ABZ, and 50 mg/kg ABZ groups, respectively. (d) Scores of the melanoma nodules in each experimental group. The scoring criteria for tumor nodule diameter were as follows: Level 1: <0.5 mm; Level 2: >0.5 mm, <1.0 mm; Level 3: >1.0 mm, <2.0 mm; and Level 4: >2 mm. (e) Representative images of HE stained mice lung sections from the control and ABZ-treated groups. The circled parts show the lung metastatic nodules. All animal experiments were repeated thrice. ^*∗∗∗*^*P* < 0.001.

Since the in vitro experiments demonstrated the inhibitory effects of low-dose ABZ on the migration and invasion of melanoma cells, we then investigated the effects of ABZ treatment on melanoma cells in vivo using a nude mouse model of lung metastasis. After 10 days of continuous intragastric administration of 20 and 50 mg/kg ABZ, which were much lower than the doses used for subcutaneous tumors [28], the in vivo fluorescence images showed that the fluorescence intensity of lung metastatic specimens from ABZ-treated mice was significantly lower than that of the control group (Figures 2(a) and 2(b)), suggesting that the lung metastasis of melanoma cells in the ABZ-treated group was greatly suppressed. In addition, the lung melanoma nodules in the ABZ treatment group were smaller and less tumors formed on the lungs (Figure 2(c)). Moreover, after the number and size of melanoma nodules were counted and scored, the nodule scores in ABZ-treated mice were discovered to be significantly lower than those in control mice (Figure 2(d)), which was confirmed by the HE staining of mice lung sections (Figure 2(e)). These results indicate that, in a dose-dependent manner, ABZ-treated mice had a significantly lower lung metastatic rate than normal. Taken together, our results demonstrate that low-dose ABZ can markedly inhibit the metastasis and invasion of melanoma cells both in vitro and in vivo.

### 3.3. ABZ Inhibits the Metastasis of Melanoma Cells by Reversing the EMT Process

**Figure 3:**
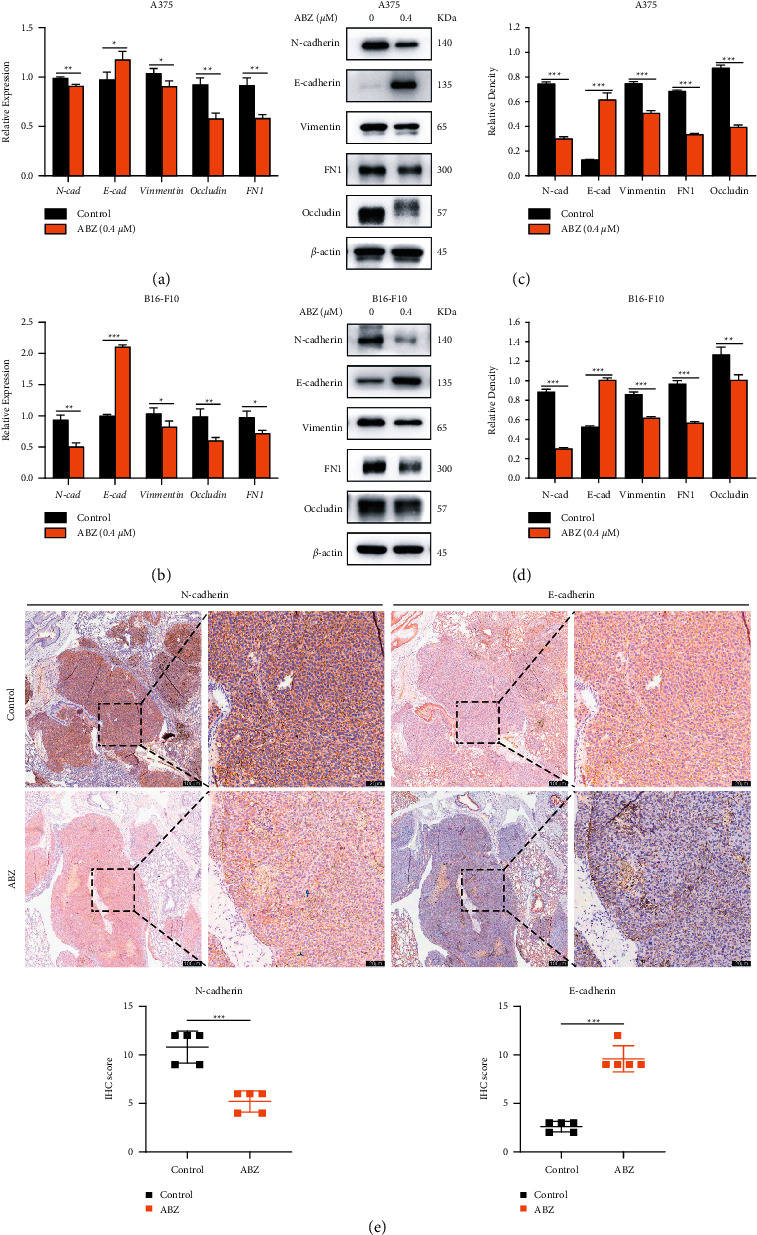
ABZ treatment inhibits the metastasis of melanoma cells by reversing the EMT process. (a-b) The relative expression levels of N-cadherin, E-cadherin, Vimentin, Occludin, and FN1 between the ABZ-treated (0.4 *μ*M) and control groups were measured by RT-qPCR, with *β*-actin as the reference gene. (c-d) The expression levels of key EMT proteins were detected by western blotting. The histogram shows the relative density of N-cad, E-cad, Vimentin, Occludin, and FN1. (e) The localization and expression levels of N-cad and E-cad in mouse lung cancer tissues were determined using an immunohistochemical (IHC) assay. Image showing the IHC staining specimens (100×; left). The black dotted box is enlarged to the area corresponding to the image (right). Scale bars = 100 and 20 *μ*m. Each point in each group in the statistical graph is calculated as the average score for five selected areas in one lung slice and represents an IHC index of one mouse. The data are expressed as means ± SD. The RT-qPCR and western blot experiments were performed thrice. ^*∗*^*P* < 0.05; ^*∗∗*^*P* < 0.01; ^*∗∗∗*^*P* < 0.001.

Since the EMT process was believed to be responsible for the metastasis and recurrence of melanoma, we also investigated the effects of ABZ treatment on the EMT process of melanoma cells. The mRNA levels of key EMT proteins, namely, N-cad, E-cad, Vimentin, Occludin, and FN1, were detected by real-time RT-qPCR analysis 24 h after ABZ treatment. Compared to the control group, *E-cad* expression was significantly upregulated in the ABZ-treated group, while the relative expression levels of N-cad, Vimentin, FN1, and Occludin were downregulated to varying degrees (Figures 3(a) and 3(b)). Furthermore, these changes were confirmed at the protein level by western blot analysis (Figures 3(c) and 3(d)). To determine the effects of ABZ on the expression of EMT-related proteins in vivo, we also analyzed the E-cad and N-cad levels in the lung sections from ABZ-treated and control mice. The IHC assay results revealed that E-cad and N-cad expression levels in the B16-F10 metastatic foci of ABZ-treated mice lung sections were significantly upregulated and downregulated, respectively, compared to the control mice (Figure 3(e)). These findings suggest that ABZ can inhibit the metastasis of melanoma cells by reversing the EMT process.

### 3.4. ABZ Downregulates the Snail Expression in Melanoma Cells by Increasing Phosphorylated GSK-3*β*/Tyr216 Accumulation

**Figure 4:**
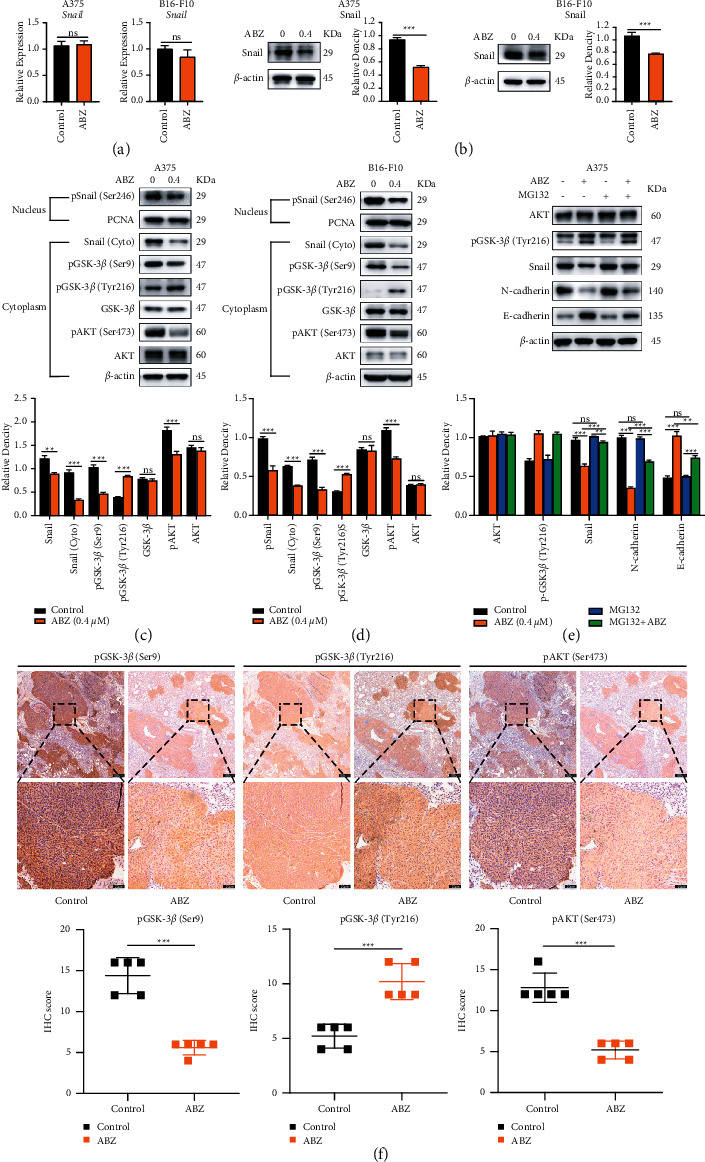
ABZ treatment downregulates the snail expression in melanoma cells by increasing the accumulation of phosphorylated GSK-3*β*/Tyr216. (a) The relative transcription levels of Snail in the ABZ-treated (0.4 *μ*M) and control groups of A375 (left) and B16-F10 (right) melanoma cells were measured by RT-qPCR, with *β*-actin as the internal control. (b) The expression of transcription factor Snail in A375 (left) and B16-F10 (right) cells was detected by western blot analysis, with *β*-actin as the internal reference protein. (c–d) The expression levels of cytoplasmic proteins AKT, pAKT, GSK-3*β*, pGSK-3*β* (Ser9/Tyr216) and Snail, and nuclear protein pSnail in A375 and B16-F10 cells were also determined by western blotting, with *β*-actin and PCNA as the internal controls for the cytoplasmic and nuclear proteins, respectively. The histograms show the relative density of AKT/pAKT, GSK-3*β*/pGSK-3*β* (Ser9/Tyr216), and Snail/p-Snail. (e) A375 cells were cotreated with or without MG132 and 0.4 *μ*M ABZ for 24 h western blot (up) was used to detect the expression levels of AKT, pGSK-3*β*/Tyr216, Snail, N-cadherin, and E-cadherin in the cytoplasm of A375 cells. The histogram (bottom) shows the relative density of AKT, pGSK-3*β*/Tyr216, Snail, E-cadherin, and N-cadherin. (f) Histogram showing the relative expression intensity of pGSK-3*β* (Ser9/Tyr216) and pAKT after immunohistochemical staining of mouse metastatic lung cancer tissues. Scale bars = 100 and 20 *μ*m. Each point in each group is calculated as the average score for five randomly selected areas in one lung slice and represents an IHC index of one mouse. All data are expressed as means ± SD. All experiments were performed thrice. ^*∗∗*^*P* < 0.01; ^*∗∗∗*^*P* < 0.001; ns, not significant.

To elucidate the molecular mechanism underlying the ABZ-mediated inhibition of the EMT process, we further investigated the expression and distribution of Snail, the main transcription factor that regulates the expression of EMT markers. The qPCR results showed that the relative expression level of Snail was not significantly altered in A375 and B16-F10 cells after ABZ treatment for 24 h (Figure 4(a)). However, ABZ treatment significantly decreased the protein levels of Snail in the cytoplasm of melanoma cells by western blot analysis (Figure 4(b)). These results suggest that ABZ potentially regulates Snail via posttranscriptional rather than translational modification. Since Snail is required to enter the nucleus to initiate transcriptional activity, we subsequently detected the level of Snail in the nucleus after ABZ treatment. As expected, ABZ treatment also reduced the levels of phosphorylated Snail/Ser 246 (pSnail/Ser246) in the nuclei of melanoma cells (Figures 4(c) and 4(d)). We also investigated the phosphorylation levels of GSK-3*β*, which negatively regulates Snail, and its upstream activator AKT [29]. Interestingly, ABZ treatment significantly reduced the levels of pGSK-3*β*/Ser9 (inactive form) and pAKT/Ser473 but greatly enhanced the accumulation of pGSK-3*β*/Tyr216 (active form) in the cytoplasm of melanoma cells (Figures 4(c) and 4(d)), and the increased ratio of Tyr216/Ser9 indicated ABZ treatment could activate GSK3*β* kinase (Supplementary Figure 1). To further confirm whether ABZ-mediated increase of pGSK-3*β*/Tyr216 could promote degradation of Snail protein in melanoma cells, MG132 was used to inhibit proteasome. Western blot analysis showed that MG132 did not affect the protein level of AKT and pGSK-3*β*/Tyr216 but significantly inhibited the degradation of Snail after ABZ treatment and subsequently resulted in the upregulation of the downstream target gene N-cadherin expression and the downregulation of E-cadherin expression (Figure 4(e)). Furthermore, the IHC results of the B16-F10 metastatic foci in mice lung sections revealed the decreased expression of pGSK-3*β*/Ser9 and pAKT/Ser473 and increased expression of pGSK-3*β*/Tyr216 in the ABZ-treated group (Figure 4(f)). Collectively, these findings indicate that ABZ can promote the nuclear export and cytoplasmic degradation of Snail by activating pGSK-3*β*/Tyr216 and inhibiting pGSK-3*β*/Ser9, resulting in the suppression of EMT progress in melanoma cells.

### 3.5. CA-AKT Significantly Reverses the Inhibitory Effects of ABZ on the Migration and Invasion of Melanoma Cells

**Figure 5:**
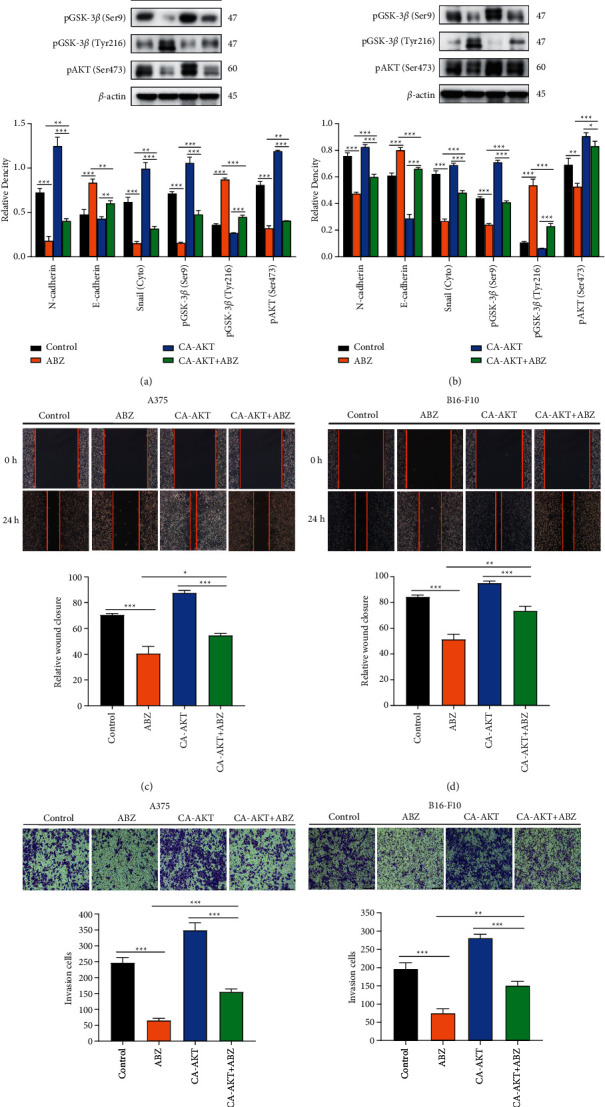
Constitutively active AKT (CA-AKT) significantly reverses the inhibitory effect of ABZ treatment on the migration and invasion of melanoma cells. (a-b) The CA-AKT plasmids were transiently transfected into A375 and B16-F10 cells, while the control group was transfected with the pcDNA3.1 (+) vector only. At 24 h after treatment with 0.4 *μ*M ABZ, western blot analysis was used to detect the expression levels of N-cadherin, E-cadherin, pAKT, pGSK-3*β* (Ser9/Tyr216), and Snail in the cytoplasm of A375 and B16-F10 cells. The histograms (bottom) show the relative density of the detected proteins. (c-d) The wound healing area and relative wound closure rate (%) in melanoma cells were quantified and analyzed 24 h after treatment with 0.4 *μ*M ABZ. The histograms (bottom) show the relative wound closure rate for each group. (e-f) Results of the transwell invasion experiments for A375 and B16-F10 cells under different conditions. The histograms (bottom) show the number of invasive cells in each group. The data are expressed as means ± SD. All experiments were performed thrice. ^*∗*^*P* < 0.05; ^*∗∗*^*P* < 0.01; ^*∗∗∗*^*P* < 0.001.

To determine the relationship between the AKT/GSK-3*β* signaling pathway and ABZ-mediated reversal of the EMT process, we transiently transfected A375 and B16-F10 cells with the CA-AKT plasmid or pcDNA3.1 (+) plasmid. Western blot analysis demonstrated that the transfection of CA-AKT elevated the protein level of pAKT/Ser473, resulting in increased pGSK-3*β*/Ser9 expression and decreased pGSK-3*β*/Tyr216 expression (Figures 5(a) and 5(b)). In contrast, CA-AKT transfection significantly reversed the ABZ-mediated downregulation of Snail in the cytoplasm, which restored the EMT progress in melanoma cells (Figures 5(a) and 5(b)). Furthermore, subsequent wound healing (Figures 5(c) and 5(d)) and transwell invasion experiments (Figures 5(e) and 5(f)) revealed that CA-AKT transfection significantly reversed the inhibitory effect of ABZ treatment on the migration and invasion of melanoma cells. These findings confirm that ABZ can suppress the EMT of melanoma cells by enhancing the pGSK-3*β*/Tyr216-mediated degradation of Snail.

### 3.6. Overexpression of GSK-3*β*/Y216F Could Not Promote the Degradation of Snail after ABZ Treatment

**Figure 6:**
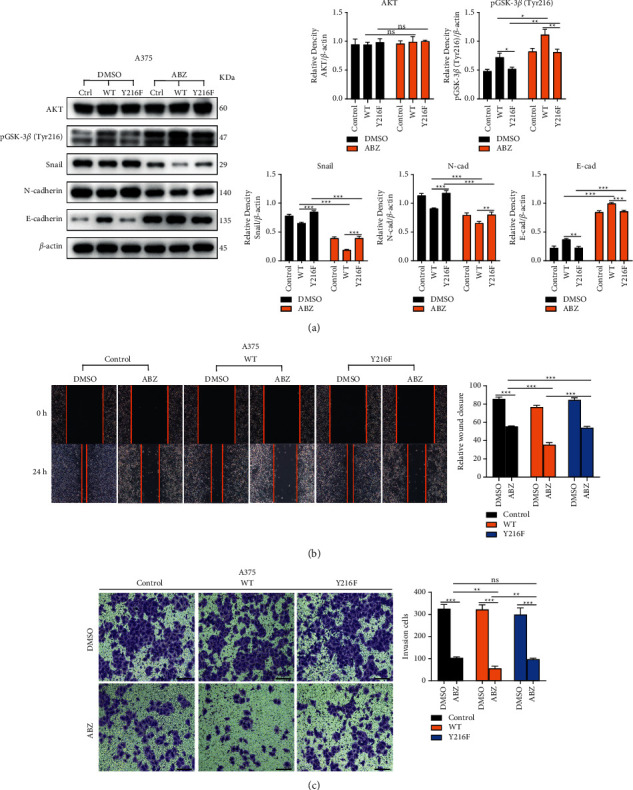
Overexpression of GSK-3*β*/Y216F could not promote the degradation of Snail after ABZ treatment. (a) Control, GSK-3*β*/WT, and GSK-3*β*/Y216F A375 cells were treated with 0.4 *μ*M ABZ for 24 h; western blot analysis (left) was used to detect the expression levels of AKT, pGSK-3*β* /Tyr216, Snail, N-cadherin, and E-cadherin in the cytoplasm. The histograms (right) show the relative density of the detected proteins. (b) The wound healing area and relative wound closure rate (%) in A375 were quantified and analyzed 24 h after treatment with 0.4 *μ*M ABZ. The histograms (right) show the relative wound closure rate for each group. (c) Results of the transwell invasion experiments for A375 cells under different conditions. The histograms (right) show the number of invasive cells in each group. The data are expressed as means ± SD. All experiments were performed thrice. ^*∗*^*P* < 0.05; ^*∗∗*^*P* < 0.01; ^*∗∗∗*^*P* < 0.001; ns, not significant.

We have demonstrated through previous experiments that ABZ can promote Snail's nuclear export and cytoplasmic degradation by inhibiting pGSK-3*β*/Ser9, thereby inhibiting the progression of EMT in melanoma cells. However, our study also revealed that ABZ enhanced the accumulation of pGSK-3*β*/Tyr216 (active form) in the cytoplasm of melanoma cells. In order to further clarify the functional role of pGSK-3*β*/Tyr216, stable A375 cell expressing wild-type GSK-3*β* (GSK-3*β*/WT) and GSK-3*β*/Y216 phenylalanine (F) mutation (GSK-3*β*/Y216F) were constructed as previous report [30]. After ABZ treatment for 24 h, the protein levels of the phosphorylation levels of GSK-3*β*/Tyr216 in the cytoplasm of all three cells were significantly increased compared with the DMSO group. Compared with ABZ-treated control cells, ABZ treatment could facilitate more GSK-3*β* phosphorylation at Tyr216 after overexpression of GSK-3*β*/WT, but not in GSK-3*β*/Y216F overexpressing cells. Meanwhile, we next detected the expression of Snail, N-cadherin, and E-cadherin proteins in three groups after ABZ treatment; as we expected, GSK-3*β*/Y216F overexpression did not change the protein levels of Snail, N-cadherin, and E-cadherin compared with ABZ-treated control cells. Furthermore, subsequent wound healing (Figure 6(b)) and transwell invasion experiments (Figure 6(c)) demonstrated that the ABZ-mediated inhibitory effect on invasion and migration of A375 cells became more pronounced in GSK-3*β*/WT overexpression cells; however, GSK-3*β*/Y216F overexpression cannot improve this effect. These results confirmed the critical role of pGSK-3*β*/Tyr216 in the EMT process of melanoma cells, suggesting that upregulation of pGSK-3*β*/Tyr216 inhibits the EMT process of melanoma cells. It was also proved that ABZ can promote the nuclear output and cytoplasmic degradation of Snail by increasing the accumulation of pGSK-3*β*/Tyr216, thus inhibiting the EMT process of melanoma cells.

## 4. Discussion

Melanoma is one of the most dangerous skin cancers responsible for >90% of patient mortality [31], especially after metastases occur, which results in the rapidly shortened survival period and very poor prognosis of melanoma patients [32]. Therefore, inhibiting metastasis progression is the main focus of melanoma treatment [31]. There is increasing evidence that benzimidazole can be used as a source of antitumor agents as a repositioned drug for cancer therapeutics [22]. As the most well-studied antiparasitic agent, ABZ has already been validated in a few tumor models [22]. However, no reports are available regarding the effect of ABZ on the EMT process of melanoma. In the present study, our results showed that low-dose ABZ treatment significantly downregulated the expression of N-cadherin, Vimentin, Occludin, and FN1 and upregulated E-cadherin expression, indicating that low-dose ABZ can effectively reverse the EMT process of melanoma cells both in vivo and in vitro.

Previous studies revealed that ABZ promotes the excessive production of reactive oxygen species in tumor cells [24], leading to high oxidative stress, Bax activation, and cell apoptosis [33] and thereby inhibiting the proliferation of tumor cells [34]. Moreover, ABZ inhibits the migration capability of pancreatic cancer cells [28] and HIF-1*α*-dependent glycolysis in lung cancer cells [35]. Interestingly, the dose of ABZ used in previous in vivo experiments was very high (e.g., 300 mg/kg twice daily [28]), which raised concerns about potential cell toxicity and adverse side effects. In this study, our findings revealed that 20 mg/kg ABZ (orally once daily) remarkably reduced the number of metastatic foci of melanoma cells in the lungs, suggesting that low-dose ABZ can effectively inhibit tumor metastasis in mice without the expected toxicity and side effects. Furthermore, results of our in vitro experiments indicated that low-dose ABZ (0.4 *μ*M, much lower than 2 *μ*M [33]), which had no effect on proliferation, significantly inhibited the migration and invasion capabilities of melanoma cells. Taken together, our in vitro and in vivo experimental data demonstrate that low-dose ABZ may be a promising intervention strategy for preventing the relapse and metastasis progression of melanoma.

Snail, an important transcription factor in the EMT process, has been observed to be highly expressed during the malignant development and progression of melanoma [36, 37]. Therefore, the regulation of Snail expression was hypothesized to be an effective strategy for controlling and preventing melanoma metastasis. The AKT/GSK-3*β* signaling pathway is one of the main survival pathways of tumor cells [38] with abnormally high activation in many malignant tumors; specifically, its dysregulation is closely related to the occurrence, migration, and invasion of malignant tumors [39, 40]. Several studies revealed that pGSK-3*β*/Ser9 (inactive form) is significantly increased in the mouse epidermal carcinogenesis model, while pGSK-3*β*/Tyr216 (active form) is markedly reduced [41, 42], indicating that the downregulation or inactivation of GSK-3*β* occurs during skin carcinogenesis [43]. Our findings verified that ABZ treatment suppresses the EMT of melanoma in vitro and in vivo by inhibiting the AKT/GSK-3*β* pathway, resulting in decreased pGSK-3*β*/Ser9 (inactive form of GSK-3*β*) expression. Meanwhile, ABZ increased the accumulation of the active form of GSK-3*β* (pGSK-3*β*/Tyr216) in melanoma cells and changed the ratio of Tyr216/Ser9. This change in the ratio may lead to the nuclear export and degradation of Snail. Furthermore, when compared with ABZ-treated control cells, GSK-3*β*/Y216F overexpression did not change the protein levels of Snail, N-cadherin, and E-cadherin after ABZ-treated; this further confirmed that pGSK-3*β*/Tyr216 could downregulate Snail protein. However, ABZ-induced inhibitory effect on migration and invasion is not subverted completely by transfection of CA-AKT, indicating that pAKT-mediated phosphorylation of GSK-3*β* at Ser9 is not the only mechanism for phosphorylated GSK-3*β*/Tyr216 accumulation after ABZ-treated. We hypothesize that this may be associated with the altered expression of kinases that can phosphorylate GSK-3*β* at Tyr216 [44]. But the mechanisms underlying the transformation of the two GSK-3*β* forms are still unknown. Hence, the relationship between ABZ and these specific kinases will be explored in our future work.

Interestingly, GSK-3*β* appears to play different roles in various cancer types. Similar to melanoma, previous research suggested that GSK-3*β* may function as a tumor suppressor for breast cancer [45]. In contrast, some studies have suggested that GSK-3*β* may promote tumorigenesis and tumor development. For example, GSK-3*β* protein was reportedly overexpressed in human ovarian [46], colon [47], and pancreatic [48] cancers. Therefore, the ABZ-mediated inhibition of tumor migration and invasion via the AKT/GSK-3*β*/Snail pathway may only be applicable to certain types of tumors. Further, the differences in the function of GSK-3*β* in various cancer types may hinder the study of ABZ as a potentially broad-spectrum cancer metastasis inhibitor. However, for melanoma, the observed inhibitory effect of ABZ on the EMT process was significant data, and the potential clinical application of ABZ still requires further research.

## 5. Conclusion

In summary, we discovered that low-dose ABZ treatment can significantly inhibit the migration and invasion abilities of melanoma cells. We verified this through in vitro and in vivo experiments and found that ABZ potentially reduces the level of pGSK-3*β* at Ser9 by downregulating pAKT/pGSK-3*β*. Furthermore, the protein level of pGSK-3*β*/Tyr216 was significantly upregulated, which promoted Snail instability and pGSK-3*β*/Tyr216-mediated degradation, and ultimately reversed the EMT of melanoma cells. Therefore, our findings contribute novel insights on how the antiparasitic drug ABZ can function in inhibiting melanoma metastasis, validating its potential as an antimelanoma metastasis inhibitor for cancer therapeutics.

## Data Availability

The raw data supporting the conclusions of this article will be made available by the authors, without undue reservation.

## Supplementary Materials

Supplementary Materials Supplementary Table 1: the primers list for RT-qPCR. Supplementary Figure 1: relative ratio changes of pGSK-3*β*/Tyr216 and pGSK-3*β*/Ser9 in A375 and B16-F10 cells after ABZ treatment.
