# Supplementary material for: Low-Dose Albendazole Inhibits Epithelial-Mesenchymal Transition of Melanoma Cells by Enhancing Phosphorylated GSK-3β/Tyr216 Accumulation
Source: J Oncol. 2021 Dec 20;2021:4475192. doi: 10.1155/2021/4475192 (PMC8712124; doi:10.1155/2021/4475192)
Supplement: Supplementary Materials — Supplementary Table 1: the primers list for RT-qPCR. Supplementary Figure 1: relative ratio changes of pGSK-3β/Tyr216 and pGSK-3β/Ser9 in A375 and B16-F10 cells after ABZ treatment. [file 4475192.f1.zip › 4475192.f1/Supplementary Figure.docx]

**Supplementary Figure**


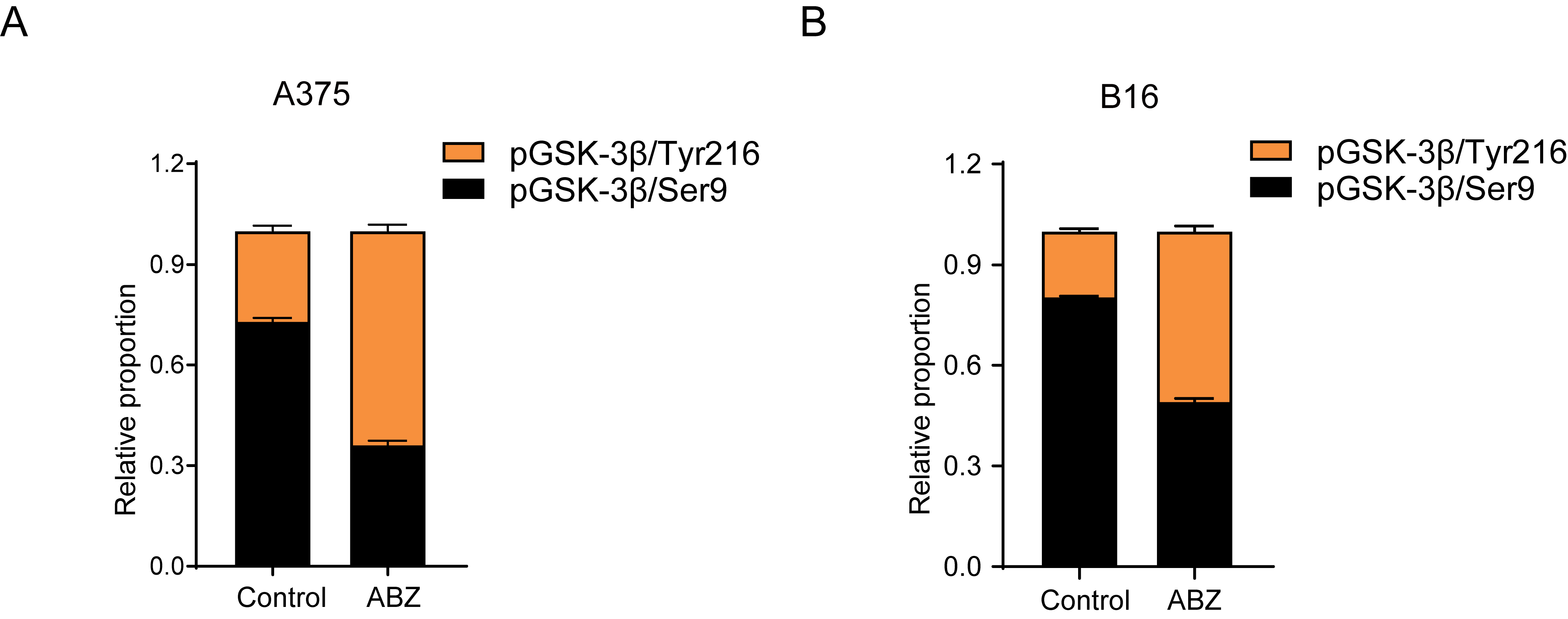


**Supplementary Figure 1 Relative ratio changes of pGSK-3β/Tyr216 and pGSK-3β/Ser9 in A375 and B16-F10 cells after ABZ treatment.**

Relative density of different form of phosphorylated GSK-3β in control group and ABZ-treated group were analyzed based on bands in Figure 4C-D, and the proportion is calculated dividing the gray value of pGSK-3β/Tyr216 or pGSK-3β/Ser9 by the total gray value of two forms. (A) The relative ratio changes of pGSK-3β/Tyr216 and pGSK-3β/Ser9 in A375 cells. (B) The relative ratio changes of pGSK-3β/Tyr216 and pGSK-3β/Ser9 in B16-F10 cells.
