# Supplementary material for: Low-Dose Albendazole Inhibits Epithelial-Mesenchymal Transition of Melanoma Cells by Enhancing Phosphorylated GSK-3β/Tyr216 Accumulation
Source: J Oncol. 2021 Dec 20;2021:4475192. doi: 10.1155/2021/4475192 (PMC8712124; doi:10.1155/2021/4475192)
Supplement: Supplementary Materials — Supplementary Table 1: the primers list for RT-qPCR. Supplementary Figure 1: relative ratio changes of pGSK-3β/Tyr216 and pGSK-3β/Ser9 in A375 and B16-F10 cells after ABZ treatment. [file 4475192.f1.zip › 4475192.f1/Supplementary Table 1-2021.8.8.docx]

Supplementary Table 1

The primer sequences of RT-qPCR.

(1).Human

| Targets | Sequences (5′-3′) |
| --- | --- |
| N-cadherin | Forward: 5’-TCAGGCGTCTGTAGAGGCTT-3’ |
|  | Reverse: 5’-ATGCACATCCTTCGATAAGACTG-3’ |
| E-cadherin | Forward: 5’-CGAGAGCTACACGTTCACGG-3’ |
|  | Reverse: 5’-GGGTGTCGAGGGAAAAATAGG-3’ |
| Snail | Forward: 5’-TCGGAAGCCTAACTACAGCGA-3’ |
|  | Reverse: 5’-AGATGAGCATTGGCAGCGAG-3’ |
| Occludin | Forward: 5’-ACAAGCGGTTTTATCCAGAGTC-3’ |
|  | Reverse: 5’-GTCATCCACAGGCGAAGTTAAT-3’ |
| Vimentin | Forward: 5’-CTTTGTCGTTGGTTAGCTGGT-3’ |
|  | Reverse: 5’-CTTTGTCGTTGGTTAGCTGGT-3’ |
| FN1 | Forward: 5’-CGGTGGCTGTCAGTCAAAG-3’ |
|  | Reverse: 5’-AAACCTCGGCTTCCTCCATAA-3’ |
| β-actin | Forward: 5’-CATGTACGTTGCTATCCAGGC-3’ |
|  | Reverse: 5’-CTCCTTAATGTCACGCACGAT-3’ |

(2).Mouse

| Targets | Sequences (5′-3′) |
| --- | --- |
| N-cadherin | Forward: 5’-ACGGACAAAGATCAGCCCC-3’ |
|  | Reverse: 5’-CTGTGACTAGCCCATCATTGCT-3’ |
| E-cadherin | Forward: 5’-AGAGTCGAAGTGCCCGAAGA-3’ |
|  | Reverse: 5’-GTGTCCCTCCAAATCCGATA -3’ |
| Snail | Forward: 5’-TCTGAAGATGCACATCCGAGC-3’ |
|  | Reverse: 5’-TTGCAGTGGGAGCAGGAGAAT-3’ |
| Occludin | Forward: 5’-TGAAAGTCCACCTCCTTACAGA-3’ |
|  | Reverse: 5’-CCGGATAAAAAGAGTACGCTGG -3’ |
| Vimentin | Forward: 5’-CAGCAGTATGAAAGCGTGGC-3’ |
|  | Reverse: 5’-GCAGGGCATCGTTGTTCCG-3’ |
| FN1 | Forward: 5’-ATCACCCTGTATGCTGTCACT-3’ |
|  | Reverse: 5’-GTGTCCCTCCAAATCCGATA-3’ |
| β-actin | Forward: 5’-GTGACGTTGACATCCGTAAAGA-3’ |
|  | Reverse: 5’-GCCGGACTCATCGTACTCC-3’ |
